# Correlative cryo-fluorescence and cryo-scanning electron microscopy as a straightforward tool to study host-pathogen interactions

**DOI:** 10.1038/srep18029

**Published:** 2015-12-10

**Authors:** Martin Strnad, Jana Elsterová, Jana Schrenková, Marie Vancová, Ryan O. M. Rego, Libor Grubhoffer, Jana Nebesářová

**Affiliations:** 1 Institute of Parasitology, Biology Centre of the Czech Academy of Sciences, Branišovská 31, České Budějovice CZ-37005, Czech Republic; 2 Faculty of Science, University of South Bohemia in České Budějovice, Branišovská 1760, České Budějovice CZ-37005, Czech Republic; 3 Department of Virology, Veterinary Research Institute, Brno CZ-62100, Czech Republic; 4 Faculty of Science, Charles University in Prague, Viničná 1594/7, Praha CZ-12800, Czech Republic

## Abstract

Correlative light and electron microscopy is an imaging technique that enables identification and targeting of fluorescently tagged structures with subsequent imaging at near-to-nanometer resolution. We established a novel correlative cryo-fluorescence microscopy and cryo-scanning electron microscopy workflow, which enables imaging of the studied object of interest very close to its natural state, devoid of artifacts caused for instance by slow chemical fixation. This system was tested by investigating the interaction of the zoonotic bacterium *Borrelia burgdorferi* with two mammalian cell lines of neural origin in order to broaden our knowledge about the cell-association mechanisms that precedes the entry of the bacteria into the cell. This method appears to be an unprecedentedly fast (<3 hours), straightforward, and reliable solution to study the finer details of pathogen-host cell interactions and provides important insights into the complex and dynamic relationship between a pathogen and a host.

*Borrelia burgdorferi* is a pathogenic bacterium whose clinical manifestations are associated with Lyme disease. This vector-borne disease is one of the major public health concerns in Europe and North America and leads to severe arthritic, cardiovascular and neurological complications if left untreated^1^. Although *B. burgdorferi* is primarily referred to as an extracellular pathogen, studies performed *in vitro* have revealed its invasive properties. It has been reported that *B. burgdorferi* is able to invade various nonphagocytic mammalian cells in order to reach an immunoprotected niche^2^,^3^.

Correlative light electron microscopy (CLEM) is a method that bridges the gap between light and electron microscopy. A limitation of fluorescence microscopy (FM) rests in the lack of structural information about cellular architecture together with the impossibility of precisely identifying unlabeled structures. Besides, resolution of conventional optical microscopes is limited to approximately 200 nm due to light diffraction^4^. Electron microscopy (EM), on the other hand, is not suitable for screening larger sample areas and is not able to provide any data about cell dynamics. The examination of the object of interest provided by CLEM offers important complementary and unique information.

Preparation of biological specimens for CLEM is a time-consuming operation, especially for the most common combination strategy; correlative FM and transmission electron microscopy (TEM). After light microscopy examination at ambient temperature, the fluorescent markers have to be subsequently labeled with immunogold and the sample has to be chemically fixed, sequentially dehydrated by alcohols, embedded in resin, polymerized, and sectioned^5^. A possible alternative, depending on the size of the sample, is to vitrify the biological specimen by rapid plunging into liquid ethane/propane or by high pressure freezing with subsequent cryo-sectioning^6^. The principal benefit of the latter strategy is that the specimen remains in a hydrated state in glass-like amorphous ice, free of preparation artifacts caused by chemical fixation, dehydration, and embedding steps^7^. These artifacts can also negatively influence the precise localization of the object of interest in the electron microscope.

A fundamental leap towards direct, fully artifact-free CLEM has been made possible by the introduction of a cryo-fluorescence microscope^8^,^9^. In order to observe exactly the same instance without any minor modification in the structure when using the light microscope and the electron microscope, it is crucial to perform fluorescence microscopy after cryo-fixation^10^. Unlike procedures where the object of interest is studied by FM before vitrification, the all-cryo-workflow (cryo-CLEM) obviates all structural or positional changes that may occur during the period between fluorescence observation and cryo-immobilization, or during the transfer of the sample to the electron microscope^11^.

Cryo-CLEM enables direct correlation of the sample in a native state and therefore it allows one to identify and visualize features that would otherwise remain hidden. Moving the realm of fluorescence microscopy closer to cryogenic applications, traditionally held by electron microscopy, is also motivated by the fact that the fluorescent molecules experience reduced photobleaching at very low temperatures^8^,^12^. There is a dual explanation for this phenomenon; reactive molecules such as oxygen or water are frozen and cannot diffuse^13^ and transformational changes of fluorophores are reduced^14^. With regard to fluorescent tags, cryo-CLEM is particularly suitable for correlating membrane permeable dyes and gene fusion proteins such as green fluorescent protein (GFP)^15^.

The goal of this study is to provide a very fast microscopy procedure (Fig. 1) that is highly convenient for high-resolution studies of pathogen-host cell interactions in a native state. Using this novel correlative cryo-FM and cryo-SEM approach, we sought to further define the cell binding properties of *B. burgdorferi* and the suggested propensity of *B. burgdorferi* towards invasion of nonphagocytic cells.

## Methods

### Bacterial strain

*B. burgdorferi* Bb914, a GFP-expressing virulent derivative of strain 297^16^ was used. *B. burgdorferi* were grown in prepared Barbour-Stoenner-Kelly (BSK-II) medium containing 6% rabbit serum (Sigma-Aldrich) at 34 °C until mid-log phase (~1 × 10^7^ spirochetes/ml), and enumerated with a Petroff-Hausser counting chamber using a dark-field microscope.

### Mammalian cell cultures

Human neural cell line UKF-NB-4 was established from bone marrow metastasis harvested in a patient with Evans stage IV neuroblastoma^17^. Cells were cultured in Iscove’s modified Dulbecco’s Media (IMDM) supplemented with 10% fetal calf serum, 1% L-glutamine, and 1% antibiotic*-*antimycotic solution (Sigma-Aldrich) at 37 °C with 5% CO_2_. Mouse neuroblastoma N2a cell line^18^ was cultured in high glucose Dulbecco’s modified Eagle’s medium (DMEM) containing ultraglutamine I (Lonza) supplemented with 10% fetal bovine serum and 1% antibiotic*-*antimycotic solution (Sigma-Aldrich) at 37 °C with 5% CO_2_.

### Cell viability assay

Trypan blue staining was performed to monitor the viability of mammalian cells after incubation with *B. burgdorferi*. Neural cells grown in tissue culture flasks were washed twice with PBS, trypsinized, and enumerated. Both sets of mammalian cells (1 × 10^6^ cells) and *B. burgdorferi*, cultured to mid-log phase, were subsequently mixed in fresh cell culture medium at a multiplicity of infection (MOI) of 5, 10, and 20. After 1 and 5 hours of incubation at 34 °C, trypan blue dye (10%) was added and the viable cells were enumerated using a Bürker counting chamber. Each infection was performed in triplicate and one hundred cells were assessed for their viability. As control, cells without *Borrelia* were examined.

### *B. burgdorferi* motility assay

Approximately 1 × 10^7^ spirochetes were centrifuged at 1500 g for 10 min at 15 °C and gently resuspended in 1 mL of either DMEM or IMDM media (with all supplements mentioned above) and maintained at 34 °C to test the motility of *B. burgdorferi* in these media. The motility was monitored for 8 hrs at 1 hr intervals on a glass slide using a dark-field microscope.

### *B. burgdorferi* viability assays

1 × 10^7^ spirochetes were centrifuged at 1500 g for 10 min at 15 °C and gently resuspended in 1 mL of either BSKII or DMEM media (with all supplements mentioned above) and maintained at 34 °C to test the viability of *B. burgdorferi*. Every 1.5 hrs equal diluted volumes from both media with borrelia, were seperately plated in duplicate. This was done for 4 time points and the plates were then kept at 34 °C under anerobic conditions for 2 weeks. The colonies formed were then counted. In addition, the same number of spirochetes after centrifugation was also resuspended in 1 ml of BSKII or DMEM media and dead cell stain propidium iodide (PI) in DMSO was added to a final concentration of 60 μM/mL. The viability was monitored for 6 hrs at 1 hr intervals on a glass slide using a fluorescence microscope (Olympus BX-60 with Olympus DP71 camera). Control experiments were performed to test the applicability of PI staining for GFP-expressing *Borrelia* (Supplementary Figure S1).

### Mammalian cell-*B.burgdorferi* association assay

Neural cells were harvested using trypsin, enumerated and 1 × 10^5^ cells were seeded 12 hrs before the start of experiment on 3 mm sapphire discs (Leica Microsystems) placed on a 24-well plate and allowed to attach at 37 °C with 5% CO_2_. Prior to this, for orientation purposes, TEM finder grids (Electron Microscopy Sciences) were placed on the sapphire discs and carbon-coated to facilitate the cell re-localization (sapphire discs were UV-sterilized for 30 min after that). *B. burgdorferi* were centrifuged at 1500 g for 10 min at 15 °C and resuspended in IMDM or DMEM, depending on the mammalian cells being used. The spirochetes (1 × 10^6^) were added to the mammalian cells and incubated at 34 °C for up to 3 hrs. At the same time, nuclear stain Hoechst 33342 (Sigma-Aldrich) was added in a final concentration of 10 μg/mL. Sapphire discs with attached cells and bacteria were washed in PBS, blotted for 2 sec (each side) at room temperature and cryo-immobilized in a home-made plunge freezing device containing liquid propane.

### Cryo-CLEM examination

For cryo-FM examination, the Leica EM CryoCLEM (Leica Microsystems) set with the Leica DM6000 FS was used. After cryo-fixation, remainders of propane were blotted using pre-cooled filter paper and the sapphire discs mounted on the cartridge were loaded directly into the microscope using a cryo-transfer shuttle cooled with liquid nitrogen. For imaging, Leica HCX PL APO 50x/0.9 CLEM objective and Leica DFC310FX camera were employed.

Following cryo-FM examination, the sapphire discs left mounted on the cartridge were put back into the cryo-transfer shuttle, where the whole cartridge was transferred under vacuum into the chamber of the cryo-attachment CryoALTO 2500 (Gatan, Inc.). The chamber was heated to −95 °C *in vacuo* for 5–20 min to remove the ice contamination by sublimation and subsequently the sample was sputter coated for 40 sec with Pt/Pd before observation. The specimens were observed in FESEM JEOL 7401F (JEOL Ltd.) operated at 1 kV with a working distance around 9 mm and the stage temperature of approximately −140 °C.

Image analysis and overlays of images were carried out using LAS X software (Leica Microsystems) and Adobe Photoshop CS6. Linear adjustments of brightness and contrast performed on fluorescence microscopy images did not obscure, eliminate, or misrepresent any information present in the original images. Final figures were constructed using CorelDraw 11.

## Results

### Cell viability assay

The cytopathic effects of *B. burgdorferi* co-culturing on the well-being of neural cell lines was investigated. For the neural cell-*B.burgdorferi* association assay, the neural cells were first seeded on the sapphire discs 12 hrs before addition of *Borrelia*. Although, the doubling times of the respective cell lines in our culture conditions were not assessed, the cell viability assay was performed at various multiplicities of infection, which should cover the whole range of potential MOIs during the mammalian cell-*B.burgdorferi* association assay. In both analysed cell lines, there were no observable adverse effects on neural cells after co-culturing with *B. burgdorferi,* when compared to the cells alone controls (Table 1). Furthermore, there were no statistically significant differences in the number of dead cells at both inspected time intervals, i.e. 1 hour and 5 hours of co-incubation. These results confirmed the ability of *B. burgdorferi* to interact and associate with neural cells without causing any observable adverse effects.

### *B. burgdorferi* motility assay

Since the association assays were carried out in cell culture media, the cytotoxic effects of DMEM and IMDM with all supplements on *B. burgdorferi* had to be determined. The motility was monitored every hour for a total of 8 hrs. During the first three examinations, the overwhelming majority of spirochetes were motile, indicating good fitness. Subsequent bacterial counts revealed a steady decrease in motility with an increasing number of nonmotile spirochetes. After 8 hrs, only *Borrelia* that formed aggregates were partially motile, the rest of spirochetes showed no such signs.

### *B. burgdorferi* viability assays

Having discerned that the antibiotic/antimycotic mixture added to both IMDM and DMEM media was the possible reason for the loss of motility, we wanted to confirm whether this motility loss was because of non-viable spirochetes. We therefore used only one of the media, DMEM for this assay. The number of colonies that grew on the plates (Table 2) indicates a significant drop in the number of viable *Borrelia* at 3 hrs compared to 1.5 hrs between the DMEM medium and the BSKII. But since it is likely that the antibiotic/antimycotic mixture may have influenced the multiplication and therefore the apparent viability of the spirochetes after antibiotic exposure, we decided to carry out one more viability assay that assessed more accurately the viability *in situ*. Fluorescence microscopy enumeration of live/dead cells revealed a significantly lower increase in the number of dead borrelia among those incubated with the DMEM media (Table 3) compared to the numbers obtained by the plating of borrelia incubated in the same media.

### Mammalian cell-*B. burgdorferi* association assay

After tick transmission, *B. burgdorferi* must cope with the strong immune response of the mammalian host. One potential strategy exploited by *Borrelia* to avoid immune clearance might be to invade the host’s nonphagocytic cells^2^. To examine this assumption, the ability of *B. burgdorferi* to associate with one human and with one mouse cell line of neural origin was tested. GFP-expressing *B. burgdorferi* cells were added at MOI of 10 to the cells. On average, 1 in 5 cells was *Borrelia*-associated under this MOI. The MOI chosen was lower than in previous studies^2^,^19^ in order to test the applicability of the cryo-correlative workflow for studies of pathogens, which are able to cause an infection at very low numbers.

The co-incubation time of *Borrelia* with neural cells was set at 3 hrs for the following reasons. We experienced cell culture contamination without the addition of the antibiotic*-*antimycotic solution. The addition of the antibiotic*-*antimycotic solution, however, limited the time interval of possible mammalian cell-*B. burgdorferi* co-incubation. Although there was a significant drop in the number of viable *Borrelia* at 3 hrs indicated by plating, the *in situ* viability assay using dead cell staining revealed the first more significant increase in the number of dead cells only after three hours of co-incubation. Moreover, the motility assay has shown that the majority of spirochetes were still motile at 3 hrs and because it was found that motility is critical for infection of the mammalian host^20^, we decided to work at this time point.

**Figure 1:**
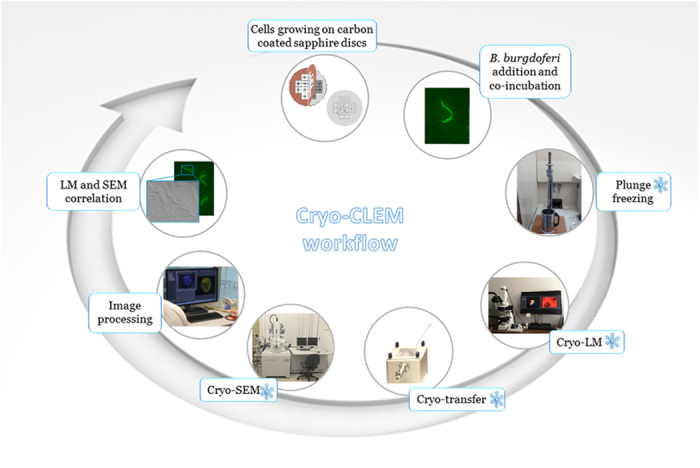
Schematic diagram illustrating the cryo-CLEM workflow.

**Figure 2:**
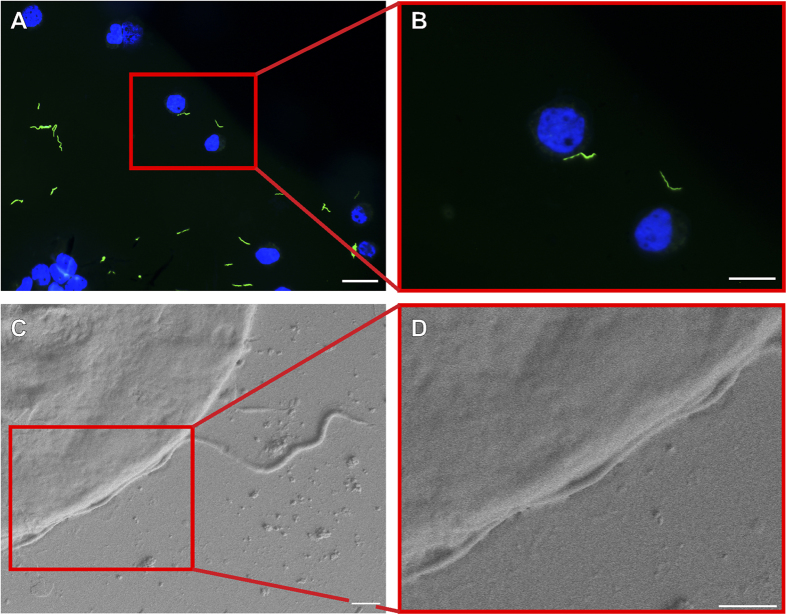
Correlative cryo-fluorescence (A,B) and cryo-scanning electron microscopy (C,D) of *Borrelia burgdorferi*-GFP on the surface of human neuroblastoma cells grown on carbon-coated sapphire discs. A series of images of one particular GFP-tagged spirochete (green) interacting with the cell counterstained with Hoechst 33342 (blue). Images of region of interest from low magnification FM to high magnification SEM. The cryo-SEM images were acquired after 10 minutes ice sublimation and deposition of Pt/Pd layer onto the sample surface. Scale bars: (**A**) 50 μm, (**B**) 25 μm, (**C**,**D**) 1 μm.

**Figure 3:**
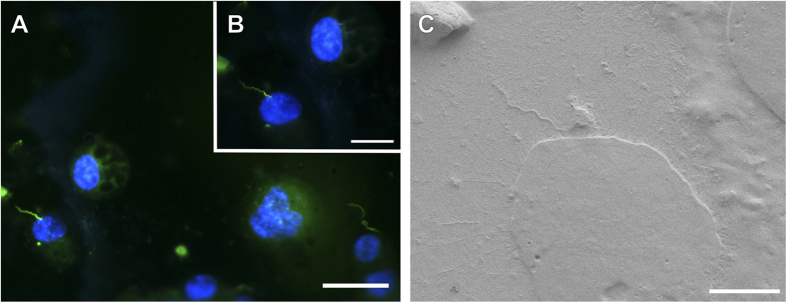
Demonstration of fluorescence preservation after deposition of Pt/Pd layer on the sample followed by SEM examination under cryo-conditions. Association of *B. burgdorferi*-GFP (green) with mouse neuroblastoma cells. Cells were counterstained with Hoechst 33342 (blue). Images acquired before (**A**) and after (**B**) cryo-SEM examination. (**B**) Image taken after 20 minutes ice sublimation and deposition of Pt/Pd layer on the sample surface. (**C**) Cryo-SEM micrograph of the spirochete-cell interaction shown in (**A**) and (**B**). Scale bars: (**A**) 50 μm, (**B**) 25 μm and (**C**) 10 μm.

Following incubation, cryo-FM was used to reveal the sparsely distributed spirochetes, which were afterwards easily located in the scanning electron microscope. The whole procedure from cryo-immobilization to SEM image acquisition (Fig. 1) lasted less than three hours, which is much less than in correlative microscopic studies at ambient temperatures. *B. burgdorferi* were observed to be associated with both cell lines examined. There were no indications suggesting that *Borrelia* can penetrate either of the cell lines within the three hour time period. We assume that *Borrelia* did not have enough time to penetrate the cells. Of course, the possibility that *Borrelia* either were inhibited by antibiotics in the cell medium, or cannot invade the cells used in this study cannot be excluded. We observed that the spirochetes retained their spiral-shape during this period of time, touching the neural cells only at a few discrete sites, which might also suggest uneven distribution of outer surface proteins important for *Borrelia*-host interplay^21^. Figures 2 and 3 show representative images of both of the cell lines interacting with *Borrelia*. It is clear from the figures that the *Borrelia* actively interact with the cells and are not idly lying on the cell surface. Low-resolution overview images from a cryo-fluorescence microscope and high-resolution details of mammalian cell-*B. burgdorferi* association acquired by cryo-SEM are displayed. The hydrated specimen was coated with a layer of Pt/Pd (2–4 nm) in the cryo-attachment chamber and observed at low kV to avoid charging artifacts and decrease sensitivy to beam damage in the microscope. Interestingly, fluorescence was not lost after covering the sample surface with a metal layer when re-examined in the cryo-fluorescence microscope (Fig. 3B).

## Discussion

For many decades, the primary method of choice for imaging host-parasite interactions has been light microscopy, especially fluorescence microscopy. However, the interplay between a pathogen and a host cell is characterized by a cascade of events occurring at the scale that are not resolved by conventional diffraction-limited FM. In order to obtain more informative ultrastructural data, some high-resolution microscopy techniques such as electron microscopy have to be co-applied^22^.

This study describes a complete cryo-correlative workflow for the localization of objects of interest by cryo-FM and their subsequent ultrastructural examination by cryo-SEM. Due to the fact that FM is performed after cryo-fixation and there are no additional procedures between cryo-FM and cryo-EM, this technique provides the ability to obtain the most precise and reliable correlative data. Using this method, one is likely to gain insight into crucial molecular mechanisms that could potentially be exploited for innovative antimicrobial strategies when looking at pathogen-host interactions.

Cryo-CLEM is a state-of-the-art approach and only few applications have been described in literature so far. In all the setups, cryo-FM was combined with cryo-electron tomography to identify GFP-tagged microtubule bundles in mammalian cells^8^, filament bundles in migrating keratinocytes^9^, mitochondria in human endothelial cells^15^, cytoplasmic bundles of MreB in *V. cholerae*^23^, cytoplasmic arrays in *R. spheroides*^24^, secretion system in *M. xanthus*^25^, and to study cell division in *Streptomyces*^26^. To the best of our knowledge, we report the first cryo-correlation between fluorescence and scanning electron microscopy. Probably the most important advantage of our setup over correlating with TEM rests in the fact that no constraints are set on the cell’s size. For TEM, cells have to grow thinner than 1 μm, better close to 500 nm, allowing them to be used for cryo-TEM without the need for cryo-sectioning^27^. Cryo-sectioning is a difficult technique that requires higher level of expertise and specialised equipment. Furthermore, correlation with SEM is ideal for study of surface interactions, which could be only hardly followed by TEM. Moreover, after cryo-FM examination, the whole cartridge holding the sapphire disc was mounted to the SEM holder. This is generally not possible for highly sophisticated holders used in electron tomography. This option is highly convenient because we avoid the possibilty of losing or damaging the sapphire disc by direct manipulation. In case of using finder grids, as it is popular practice in the cryoFM-TEM studies^24^, the quite common scenario of bending the grid is also omitted in our setup.

In our hands, the cryo-CLEM has proven to be an extremely straightforward and efficient method. Still there are few points to keep in mind. Firstly, transferring the sample from the plunge freezer into the light microscope and later on into the electron microscope has to be precisely controlled to avoid any adverse contamination and structural damage. It is advisable to maintain sample temperature below −135 °C^10^, preventing vitreous ice from crystallizing into cubic or hexagonal ice, which adversely affects the cellular ultrastructure. Secondly, what saved time during the cell of interest re-localization was keeping sapphire discs mounted on the cartridge between cryo-FM and cryo-SEM imaging, as sometimes the lettering of the finder grid pattern marked out on the sapphire discs was not clearly discernible. The cartridge clamps securing the sapphire disc from movement provided an ideal starting point for EM examination (Supplementary Figure S2). Thirdly, even with great care, slight ice contamination is usually formed on the sample surface during the sample manipulation and its transfer^28^. This obscures the visualization of structures of interest and, moreover, it enhances the charge artifacts. In order to remove the ice contamination from the sample surface, the cryo-attachment chamber was heated to −95 °C. The vacuum sublimation reduced ice contamination allowing the observation of higher structural details, but on the other hand, prolonged heating (generally >15 min) caused the formation of sublimation artifacts such as small pits in the sample surface.

After cryo-SEM examination under high vacuum (approximately 1 × 10^−5^ Pa), it was possible to locate again the object of interest in the cryo-fluorescence microcope since fluorescence was not attenuated. The resistance to fluorescence attenuation was observed with two fluorescent molecules of different colours, namely green fluorescent protein and blue nuclear dye Hoechst 33342. The potential reduction in fluorescence intensity was not quantified since not enough data had been acquired. Of note, fluorescence was not lost even after deposition of a Pt/Pd layer on the specimens in the cryo-attachment chamber of the scanning electron microscope. Preservation of Hoechst 33342 signal can be rationalized by the fact that this nuclear dye is hidden inside the cells and shielded from the metal particles. On the other hand, the resistance of GFP to fluorescence quenching can be presumably attributed to a thin layer of ice covering the GFP-expressing *B. burgdorferi.* The GFP signal was well-detectable even after 20 minutes of ice sublimation followed by metal layer deposition. Longer times of ice sublimation were not tested since after 20 minutes the cell surface structure was impaired. The observation of fluorescence preservation can have fundamental implications for instance on sample preparation for integrated light and electron microscopes^29^. It might also come in useful when an interesting object or phenomenon is spotted using the electron microscope and needs to be reassessed with the light microscope.

*B. burgdorferi* being a zoonotic pathogen finds itself in two very different environments – a mammalian host and the arthropod tick vector. Both of these environments provide enough physical/cellular barriers for the spirochete to be in motility mode or adhering to cells if not inside certain cells^30^. In the tick the *Borrelia* migrate from the midgut to the salivary glands of an infected tick upon blood feeding followed by entrance into the mammal through the tick bite site. The spirochete also enters a tick that is feeding on a reservoir host by leaving the site of tick feeding and entering the naive tick through the bloodmeal. In a mammalian host it has to disseminate from the bite site to secondary points of infection and persist within particular niches^31^. All these concerted movements in the two environments are assumed or known to involve the process of motility and adhesion to cells^32^. It has been shown that *Borrelia* initially tether to a cell followed by dragging and then extravasation^33^. These activities require the use of a complex of proteins that are being expressed on the surface of the *Borrelia*. Identification of most of the surface adhesins in *Borrelia* has been through *in vitro* assays^34^. Some of these studies have made use of either light microscopy techniques or EM to look at the actual state of adhesion. Recent studies exploring the intricacies of *B. burgdorferi* internalization by mammalian cells *in vitro* have also utilized either immunofluorescence microscopy^2^,^3^ or solely transmission electron microscopy without *Borrelia*-specific labeling^35^ for determining the extracellular or intracellular location of the spirochetes. Immunofluorescence techniques might be sometimes misleading since the relative mutual positioning of a cell and a pathogen is hard to be reliably discerned without ultrastructural information. On the other hand, the search for spirochetes over larger areas (e.g. over the 3 mm sapphire disc) using EM can take hours due to their size and their slender morphology. One can see many structures or artifacts that can resemble *Borrelia* by their shape and without specific labeling these structures can be falsely identified (Supplementary Figure S3). So that no ambiguity can arise, it is fundamental to use the correlative approach.

In summary, we have established a very fast, preparation-artifact-free and easily attainable correlative cryo-FM and cryo-SEM workflow with the intention to streamline the generally prolonged ambient temperature correlative procedures. Using this technique, we have shown that *B. burgdorferi* associates with mammalian nonphagocytic cells, but is not able to invade them within three hours of co-incubation. The discussed technique is suitable for a wide array of applications ranging from single cell studies to whole tissue examination. Especially appealing for future work appears to be the combination of cryo-super resolution FM^36^ and cryo-SEM, which could allow the organization of fluorescently labeled surface proteins to be studied with near-to-nanometer resolution.

## Additional Information

**How to cite this article**: Strnad, M. *et al.* Correlative cryo-fluorescence and cryo-scanning electron microscopy as a straightforward tool to study host-pathogen interactions. *Sci. Rep.* **5**, 18029; doi: 10.1038/srep18029 (2015).

## Supplementary Material

Supplementary Information

## Figures and Tables

**Table 1:** Cell viability assay.

**Number of viable cells following** ***B. burgdorferi*** **co-incubation**^a^				
Neural cell line	MOI	*Borrelia* (1 hr)^b^	*Borrelia* (5 hrs)^b^	No *Borrelia*control (5 hrs)
UKF-NB-4	5	100	99	98
	10	97	98	
	20	100	98	
N2a	5	91	90	90
	10	89	90	
	20	88	88	

^a^100 cells counted (average out of 3 measurements; rounded value).

^b^Cells co-incubation time with *B. burgdorferi.*

**Table 2:** *B. burgdorferi* viability assay using plating.

Time-point (hrs)	BSKII (No. of colonies)^a^	DMEM (No. of colonies)^a^
1.5	372	368
3	380	52
4.5	382	46
6	414	0

^a^average of two plates.

**Table 3:** *B. burgdorferi* *in situ* viability assay using dead staining.

Time-point (hrs)	BSKII (No. of dead cells)^a^	DMEM (No. of dead cells)^a^
1	0	0
2	–	1
3	0	2
4	–	6
5	–	12
6	0	14

–Not measured.

^a^100 cells counted (average out of 3 measurements; rounded value).
