# Supplementary material for: Correlative cryo-fluorescence and cryo-scanning electron microscopy as a straightforward tool to study host-pathogen interactions
Source: Sci Rep. 2015 Dec 10;5:18029. doi: 10.1038/srep18029 (PMC4674872; doi:10.1038/srep18029)
Supplement: Supplementary Information [file srep18029-s1.pdf]

Correlative cryo-fluorescence and cryo-scanning electron microscopy as a straightforward tool to study host-pathogen interactions

Martin Strnad*, Jana Elsterová, Jana Schrenková, Marie Vancová, Ryan O. M. Rego, Libor Grubhoffer & Jana Nebesářová

Supplementary Information

Supplementary Figure S1: Representative images of *B. burgdorferi* observed with fluorescence microscopy using dead stain. Live spirochetes express GFP (green), dead spirochetes stained with PI (red). First row, spirochetes in 1:1 mixture of BSKII culture medium and water after 15 min of incubation, justifying the applicability of PI dead staining for GFP-expressing *Borrelia*. Second row, spirochetes in BSKII medium after 6 hrs of incubation with PI, showing no adverse effects of PI stain on spirochetes. Last row, spirochetes after 3 hrs of incubation in DMEM.

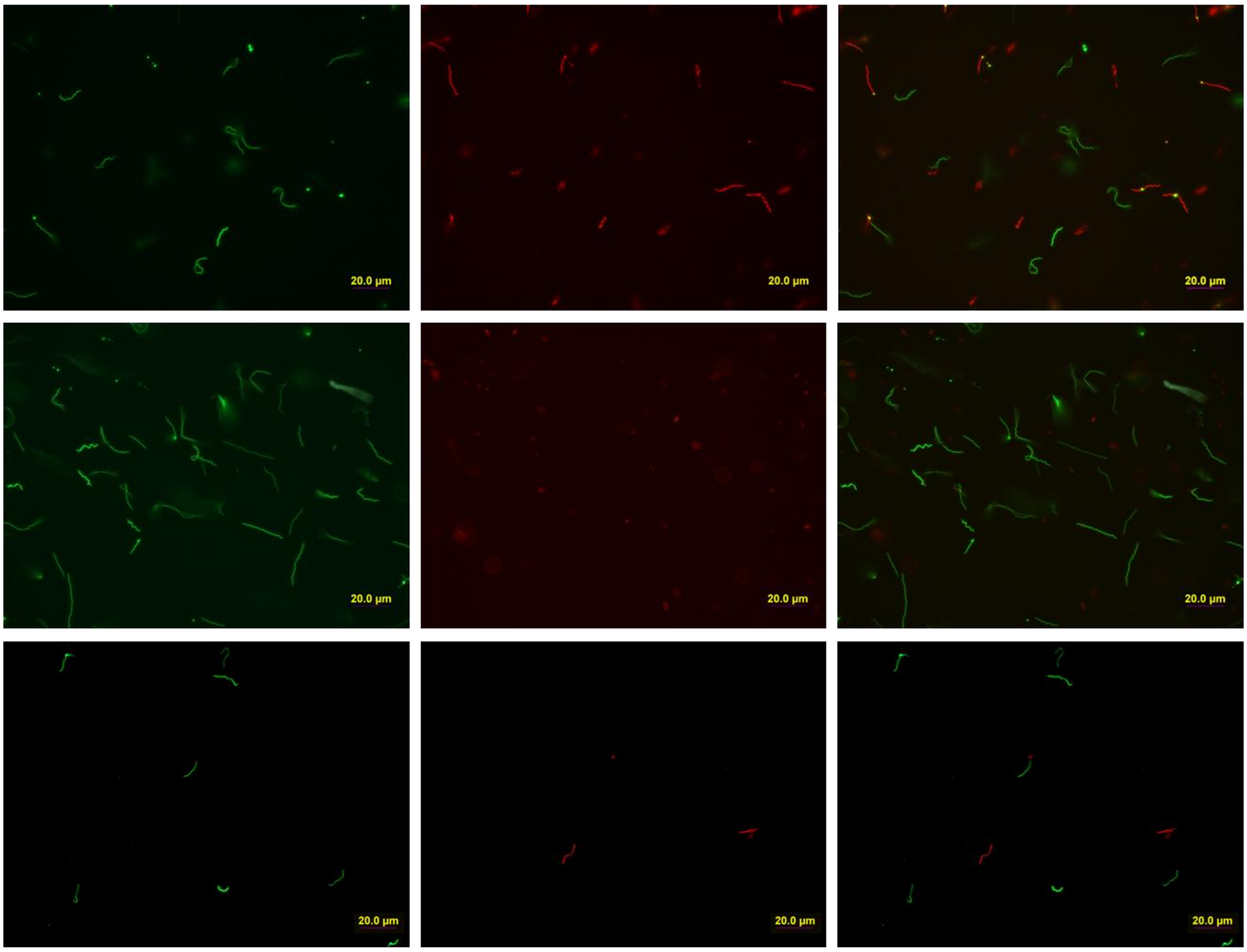

Supplementary Figure S2: The cartridge clamp (white arrow) securing the sapphire disc from movement. TEM finder grid pattern (black arrow) on the disc facilitates cell relocalization.

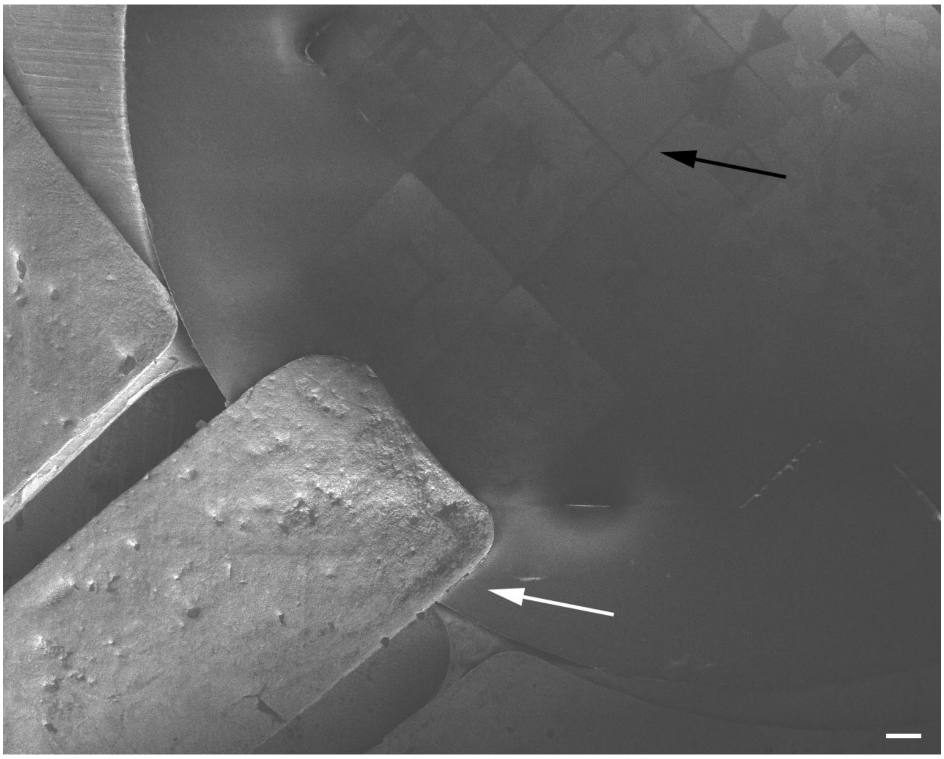

Scale bar: 100 µm.

Supplementary Figure S3: (A) The sapphire disc with the clumps of cells (circles). (B) Approximately the boxed area from (A). The structures that by their shape resemble *Borrelia* and without correlative approach could be falsely identified as *Borrelia* (arrows).

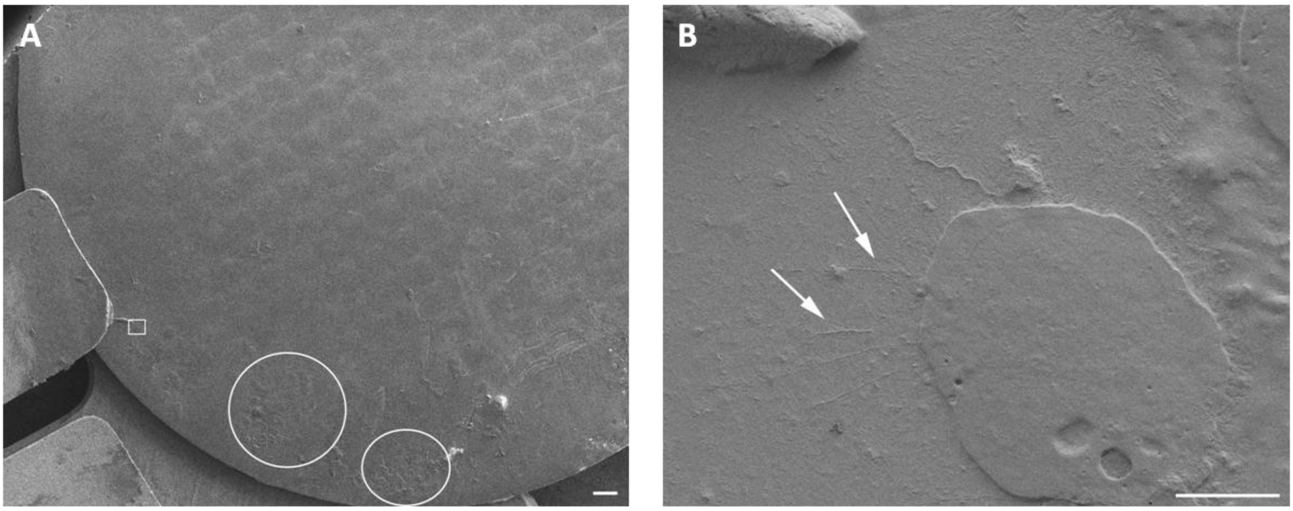

Scale bars: (A) 100 µm, (B) 10 µm.
